# Natural Killer Cell Degranulation Defect: A Cause for Impaired NK-Cell Cytotoxicity and Hyperinflammation in Fanconi Anemia Patients

**DOI:** 10.3389/fimmu.2019.00490

**Published:** 2019-03-21

**Authors:** Snehal Shabrish, Madhura Kelkar, Niranjan Chavan, Mukesh Desai, Umair Bargir, Maya Gupta, Priti Mehta, Akanksha Chichra, Chandrakala S, Prasad Taur, Vinay Saxena, Babu Rao Vundinti, Manisha Madkaikar

**Affiliations:** ^1^Department of Paediatric Immunology and Leukocyte Biology, National Institute of Iummunohematology (ICMR), KEM Hospital, Mumbai, India; ^2^Division of Immunology, Bai Jerbai Wadia Hospital for Children, Mumbai, India; ^3^Surya Hospitals, Mumbai, India; ^4^Department of Haematology, Seth G. S. Medical College and KEM Hospital, Mumbai, India; ^5^National Institute of Virology, Mumbai Unit, Haffkine Institute, Mumbai, India

**Keywords:** Fanconi anemia, NK cell degranulation defect, Familial Hemophagocytic lymphohistiocytosis (FHL), HLH-targeted therapy, NK cell cytotoxicity

## Abstract

Fanconi anemia (FA) is a rare inherited syndrome characterized by progressive bone marrow failure (BMF), abnormal skin pigmentation, short stature, and increased cancer risk. BMF in FA is multifactorial and largely results from the death of hematopoietic stem cells due to genomic instability. Also, inflammatory pathology in FA has been previously reported, however the mechanism is still not clear. In literature, decreased NK-cell count and/or impaired NK-cell activity, along with other immunological abnormalities have been described in FA-patients (1). However, to the best of our knowledge, this is the first report showing a defective degranulation mechanism leading to abnormal NK-cell cytotoxicity in FA-patients, which may explain the development of a hyperinflammatory response in these patients. This may predispose some patients to develop Hemophagocytic lymphohistiocytosis (HLH) which manifests with prolonged fever, progressive cytopenias and organomegaly. Early diagnosis and initiation of immunosuppressive therapy in these patients will help to better manage these patients. We also propose FA genes to be listed as a cause of familial HLH.

## Introduction

Fanconi anemia (FA) is a disorder of chromosomal instability caused by homozygous or compound heterozygous mutations in any of the 21 DNA repair genes of the FA/BRCA pathway (2). It is a rare autosomal recessive or X-linked disorder affecting all ethnic groups, with an incidence of 1 in every 350,000 live births (3–5). Clinically, patients have short stature, skin abnormalities, like hyper or hypopigmentation and Café-au-lait spots, various congenital defects like radial ray skeletal abnormalities, progressive bone marrow failure (BMF) and increased susceptibility to the development of hematological and epithelial malignancies. The gold standard for diagnosis of FA is the demonstration of increased chromosomal breakages after exposure to mitomycin C (MMC) and diepoxybutane (DEB) however, final diagnosis requires identification of pathogenic mutations (5, 6).

Cytopenia due to BMF are the most common presenting manifestations seen in nearly 77% of patients, whereas almost all (96%) eventually develop hematological manifestations (7). BMF in FA is multifactorial and largely results from cumulative DNA damage caused by genomic instability (8). It may occasionally result from progression to either myelodysplastic syndrome (MDS) or acute myeloid leukemia (AML). Inflammatory cytokines are shown to be elevated in patients with FA. The FA cells are also susceptible to damage by the chronic inflammatory cytokines, leading to eventual BMF (9, 10).

Various subtle immunological abnormalities including reduced T, B, and NK cell numbers, abnormal T cell proliferative responses and abnormal NK cell cytotoxicity has been reported in patients with FA (1, 11). However, to the best of our knowledge, this is the first report showing abnormal granule mediated cytotoxicity of NK cells in FA patients. This may predispose FA patients to develop Hemophagocytic lymphohistiocytosis (HLH) manifesting with prolonged fever, progressive cytopenias and organomegaly.

## Materials and Methods

### Samples

Patients (*n* = 9) diagnosed with Fanconi Anemia at the National Institute of Immunohematology (NIIH) were included in this study. Detailed clinical and family history was recorded for these patients. After obtaining informed consent, 3 mL peripheral blood was collected in EDTA, plain and heparinized vacutainers.

Age-matched healthy volunteers' blood samples (*n* = 12) were obtained as controls (seven males and five females). These individuals has no history of any febrile or other illnesses in the previous 3 months.

FHL patients with defective NK cell degranulation mechanism (*n* = 13) were included in this study as the control group.

### Diagnosis of FA

A chromosome breakage study was done on Phytohemagglutinin (PHA) stimulated lymphocyte cultures induced with Mitomycin C (MMC) at a final concentration of 40 ng/ml and incubated at 37°C for 72 h. Cells were then arrested with colchicine at the 68th h of the metaphase stage, followed by hypotonic solution treatment using 0.075 M potassium chloride, and fixation with Carnoy's fixative (3:1 methanol: glacial acetic acid). The cells were then dropped on pre-chilled slides and stained with Giemsa stain. A total of fifty metaphases were scored under a bright field microscope and chromosomal breakages and radial forms were recorded and compared with the negative control (or non-FA) sample each time (12).

Chromatid and chromosome breaks, and acentric fragments were scored as one break. Dicentric and ring chromosomes were scored as two breaks. Numbers of breaks in the radial configurations were scored as the number of chromosomes involved in the configuration. For each patient, the chromosome damage was scored as the number of breaks per cell. A score above one break per cell was considered as being fanconi anemia positive and selected for the study.

### Immunological Workup

Lymphocyte subsets NK cells, T cells, B cells, cytotoxic T cells, and helper T cells were enumerated using a dual platform. Absolute white blood cells (WBC) count and lymphocyte absolute count was determined using Sysmex XS-800i. Lymphocyte subset analysis by flow cytometry using BD Multitest 6-color TBNK reagent followed by acquisition of cells on FACSAria fusion; analysis was performed on FACS Diva 8.0 (BD Biosciences, San Jose, CA, USA).

For intracellular Perforin staining, cells were fixed and permeabilized with cytofix/cytoperm kit (Becton Dickinson) and stained with perforin-PE (Gδ9) as previously described (13).

For the granule release assay, cells were stimulated with Phorbol-12- myristate-13-acetate (PMA, 0.15 μg/ml, Sigma Chem. Co., St. Louis, MO) and Ca^2+^ Ionophore (Ionomycin, 3 μg/ml, Sigma Chem. Co., St. Louis, MO) for 2 h and CD107a-FITC (H4A3) expression (degranulation marker) was determined as previously reported in literature (14)

Lymphocytes were gated on forward and side scatter parameters and NK cells were gated as CD56^+^CD3^−^. The results were expressed as the percentage of cells in a gated NK cells region. At least 20,000 lymphocytes were acquired on FACSAria fusion cytometer (Becton Dickinson) and was analyzed using FACS DIVA software.

The NK-cell cytotoxicity was determined using a flow cytometry based assay as published previously (15). The target cells (K562 cells) were labeled with DIOC18 dye (fluorescent dye) (Sigma Aldrich) and co-incubated with effector cells at different ratios (50:1, 100:1, 200:1). 7-AAD (viability dye) (BD Pharmingen, San Diego, CA) was used to detect the dead target cells. Serum samples were separated from peripheral venous blood and were stored at −80°C for further use. Serum levels of 14 different cytokines (IL-2, IL-4, IL-6, IL-7, IL-10, IL-15, IL12p40, IFN-γ, TNF-α, GM-CSF, MIP1a, MIP1b, hMCP-1, and IP-10/CXCL10) in five FA patients, 13 HLH patients and 12 healthy controls were determined by flow cytometry using an AIMPLEX kit (Aimplex Biosciences, Inc.) as per the manufactures instructions. The assay is based on the principle of sandwich ELISA. In the AimPlex multiplex assay, multiple bead populations differentiated by size and different levels of fluorescence intensity are coated with capture antibodies specific for different cytokines. The assay procedure consists of a 60 min antigen and capture antibody-conjugated bead incubation step, a 30 min biotinylated antibody incubation and detection step, and a 20 min streptavidin–phycoerythrin incubation step. The samples were acquired on the FACSAria fusion cytometer (Becton Dickinson) to measure the fluorescence signal of the sample beads, and the results were analyzed using the FCAP Array software V3.0 (BD Biosciences, San Jose, CA, USA).

### Viral Infection Detection

All patients were screened for nine different viral infections viz. human adenovirus, human cytomegalovirus, Epstein-Barr virus, herpes simplex virus 1 and 2, varicella zoster virus, enterovirus, human parechovirus, human herpes virus 6 and 7, human parvovirus B19 using multiplex Real-Time PCR technique (FTD Neuro9 kit, Fast Track Diagnostics Luxembourg).

### Molecular Investigation

Molecular investigations in these patients were done by targeted gene capture using a custom capture kit by Medgenome Labs Pvt Ltd, India. The libraries were sequenced on an Illumina sequencing platform (mean coverage >80 to 100X). The identified mutations were confirmed by Sanger sequencing.

### Statistical Analysis

Median and range for all the parameters was calculated. A 2-way ANOVA test and Mann-Whitney U test was used to compare data between the groups. The Spearman correlation was performed to analyze the correlation between variables. Analyses were performed with the GraphPad Prism (GraphPad Software, Inc. Version 5.0). Differences were considered statistically significant if the 2-tailed *p*-value was ≤ 0.05.

## Results

### Clinical Laboratory and Molecular Findings

Patient P1, a 3-year-old female child born from a 3rd degree consanguineous marriage, was referred to us with a fever of unknown origin that lasted for more than 15 days, cytopenia and hepatosplenomegaly to rule out primary HLH. The patient fitted into the HLH criteria (16) with elevated serum ferritin levels (12,000 ng/ml). The workup for primary HLH included flowcytometric analysis of intracellular perforin expression on NK cells and a degranulation assay. Perforin expression on NK cells was normal (75%; reference range 72 ± 2%); however, the granule release assay (GRA) was abnormal (6.5%; reference range 28 ± 8%) suggesting a defect in the granule release mechanism leading to HLH. Along with these clinical manifestations, the patient had short stature and a bifid right thumb; in view of these phenotypical features, Fanconi anemia's work up was performed which showed a mitomycin (MMC) induced high frequency of chromosomal breakages. As the patient was affected with both FHL and FA, NGS-clinical exome panel was performed to identify the underlying genetic defect, which revealed a homozygous mutation in the *FANCA* gene (c.2368delC; p.His790ThrfsTer33) and no mutation was identified in HLH-related genes.

Following this observation, eight FA patients were tested for GRA. Of these, P2 also fitted into the HLH criteria. In these nine FA patients, four had anemia, eight had thrombocytopenia and only four had neutropenia. Clinical and laboratory parameters of all these patients are given in Table 1.

**Table 1:** Clinical and laboratory findings in FA patients.

	**P1**	**P2**	**P3**	**P4**	**P5**	**P6**	**P7**	**P8**	**P9**
Age (years)	3	15	4	15	3	12	4.5	8	3
Sex	Female	Male	Female	Female	Male	Female	Female	Male	Female
Fever >7 days	**Y**	**Y**	**Y**	**Y**	**Y**	**N**	**N**	**Y**	**N**
Hepatosplenomegaly	**Y**	**Y**	**Y**	N	N	**N**	**N**	**N**	N
Bicytopenia	**Y**	**Y**	N	N	N	**Y**	**Y**	N	N
Hb	**9.5**	**6.2**	10.6	10.2	10.7	**8.6**	**6.8**	9.8	12
Neutrophil counts (x10^3^ cells/mm^3^)	**0.66**	2.66	2.12	1.25	1.0	**0.92**	**0.72**	**0.67**	2.11
Lymphocyte counts (x10^3^ cells/mm^3^)	2.67	1.49	3.06	1.76	4.13	1.07	3.16	3.44	7.29
Platelets Counts (x10^3^ cells/mm^3^)	**32**	**33**	**63**	**93**	176	**26**	**21**	**18**	**77**
Triglyceride (mg/ml)/ Fibrinogen (mg)	177/325	111.8/**118**	ND	ND	ND	ND	ND	ND	ND
Hemophagocytosis	**Y**	**Y**	N	N	**Y**	**N**	**N**	**N**	**N**
NK cell activity	**Low**	ND	**Low**	ND	**Low**	**Low**	**Low**	**Low**	**Low**
Ferritin (ng/ml)	**12000**	**18200**	51.3	455	**642**	**799**	115	**643**	35
sCD25 (pg/ml)	3745	3187	2869	2468	3126	3629	2536	3244	3112
HLH Criteria fulfilled	**6/8**	**6/8**	2/8	1/8	3/8	3/8	2/8	3/8	1/8
Fits into HLH criteria (16)	**Y**	**Y**	N	N	N	N	N	N	N
**Breaks/cell** (reference range: 0.04–0.06)	3.67	3.80	3.70	4.09	3.70	3.8	2.15	1.17	3.2
**FA gene involved**	*FANCA*	*FANCA*	*FANCG*	*FANCN*	*FANCL*	Awaited	Awaited	Awaited	Awaited
**Mutation**	c.2368delC (p.His790Thrfs Ter33)	c.3215_3218delAGAG (p.Gln1072ArgfsTer4)	c.787C>T (p.Q263X)	c.560C>A (p.Pro187His). c.1739A>G (p.Tyr580Cys)	c.1107G>A (p. Lys369 Lys)				
**Zygocity**	Homozygous	Homozygous	Homozygous	Compound heterozygous	Homozygous				
**Novelty**	Novel	Novel	Reported	Reported/Novel	Reported				

*Y, Yes; N, No; ND, Not Done. Bold words indicate positive parameters for diagnosis of HLH*.

### Chromosomal Breakage Studies

All subjects showed a significantly high frequency of chromosomal breakage (breaks/per metaphase) compared to the controls, which confirmed the diagnosis of FA (Table 1; Supplementary Figure 1).

### Immunological Findings

Lymphocyte subset analysis in these patients revealed low absolute counts of NK cells in seven patients, except for two patients (P8 and P9). T cell and B cell absolute counts were within normal range except for patients P6 and P7–8, respectively (Table 2).

**Table 2:** Immunological findings in FA patients.

	**P1**	**P2**	**P3**	**P4**	**P5**	**P6**	**P7**	**P8**	**P9**
NK cell frequency (%)	2	3.3	2.3	1	1.6	2.5	3	4	8
NK cell numbers (cells/mm^3^)	**53** ^L^	**49** ^L^	**70** ^L^	**18** ^L^	**66** ^L^	**27** ^L^	**95** ^L^	138	584
T cell frequency (%)	70	69	75	78	90	82	89	89	67
T cell numbers (cells/mm^3^)	1,869	1,028	2,295	1,373	3,717	**878**^L^	2,812	3,065	4,888
B cell frequency (%)	25	28	22	17	8	10	7	5	24
B cell numbers (cells/mm^3^)	668	417	673	299	330	107	**221** ^L^	**172** ^L^	1751
Perforin expression (%) (reference range: 72 ± 8%)	75	67	78	72	92	96	73	88	92
CD107a expression on stimulated NK cells [Granule release assay (GRA)] (%)(Reference range: 28 ± 8%)	**6.5** ^L^	**10** ^L^	**14** ^L^	**9** ^L^	**12** ^L^	**8.2** ^L^	**9.7** ^L^	**7.4** ^L^	**9.7** ^L^

L *, lower than the reference range*.

^*^ Normal ranges reference (17).

*Bold values indicate positive parameters for diagnosis of HLH*.

**Figure 1:**
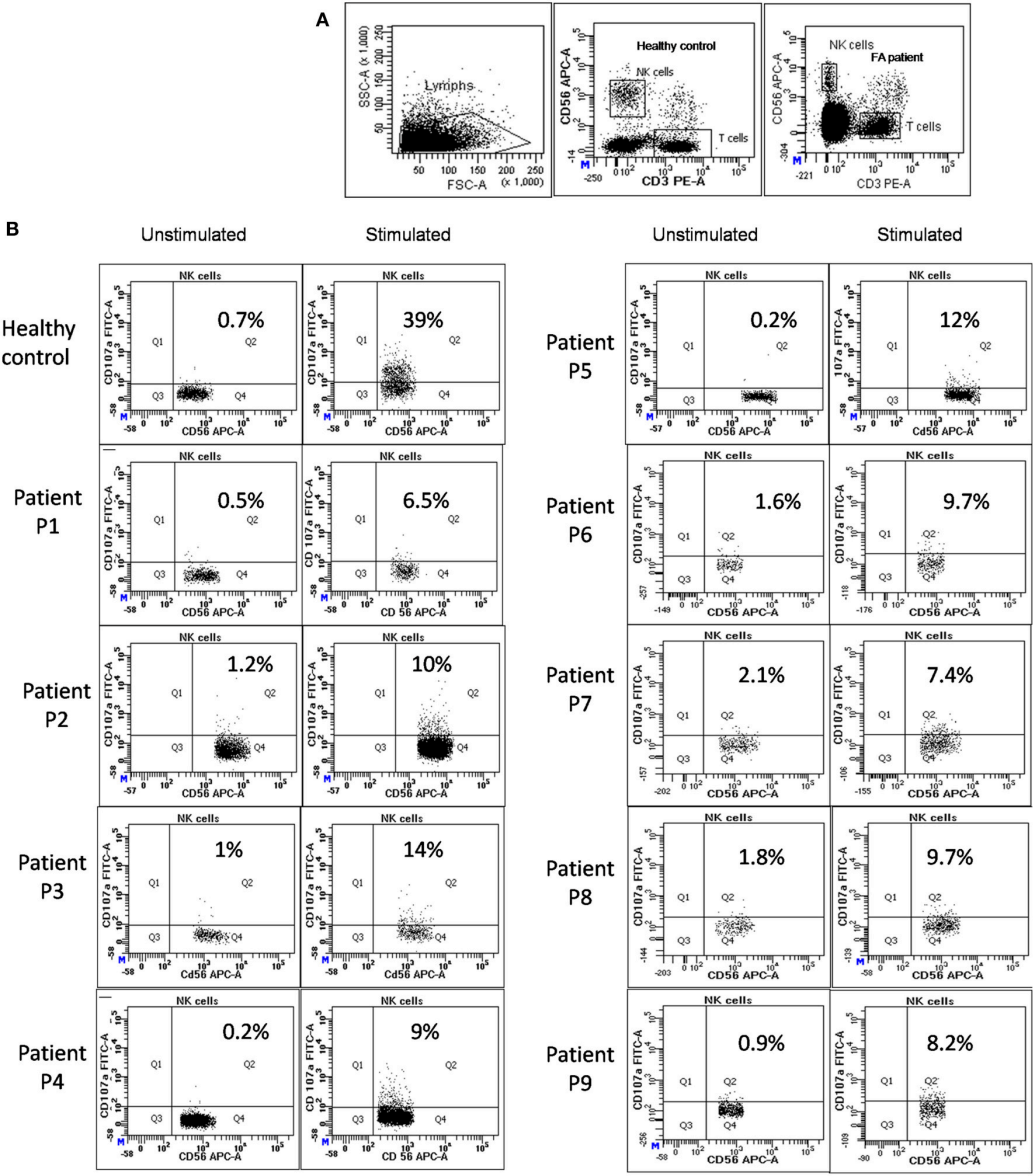
NK cell degranulation assay results in FA patients: **(A)** Samples were analyzed by flow cytometry, gating on lymphocytes by forward/side scatter. CD107a expression was analyzed on natural killer (NK) cells (CD56+CD3-) **(B)** Degranulation assay results from a healthy control are shown (representative plot) and from the patients with FA included in this study. The percentage of CD107a+ NK cells is indicated on each plot. In the healthy controls the CD107a expression on NK cells increased significantly after Ca-I and PMA stimulation. However, in FA patients CD107a expression on stimulated NK cells was significantly lower than the healthy controls, indicating defective granule release mechanism.

**Figure 2:**
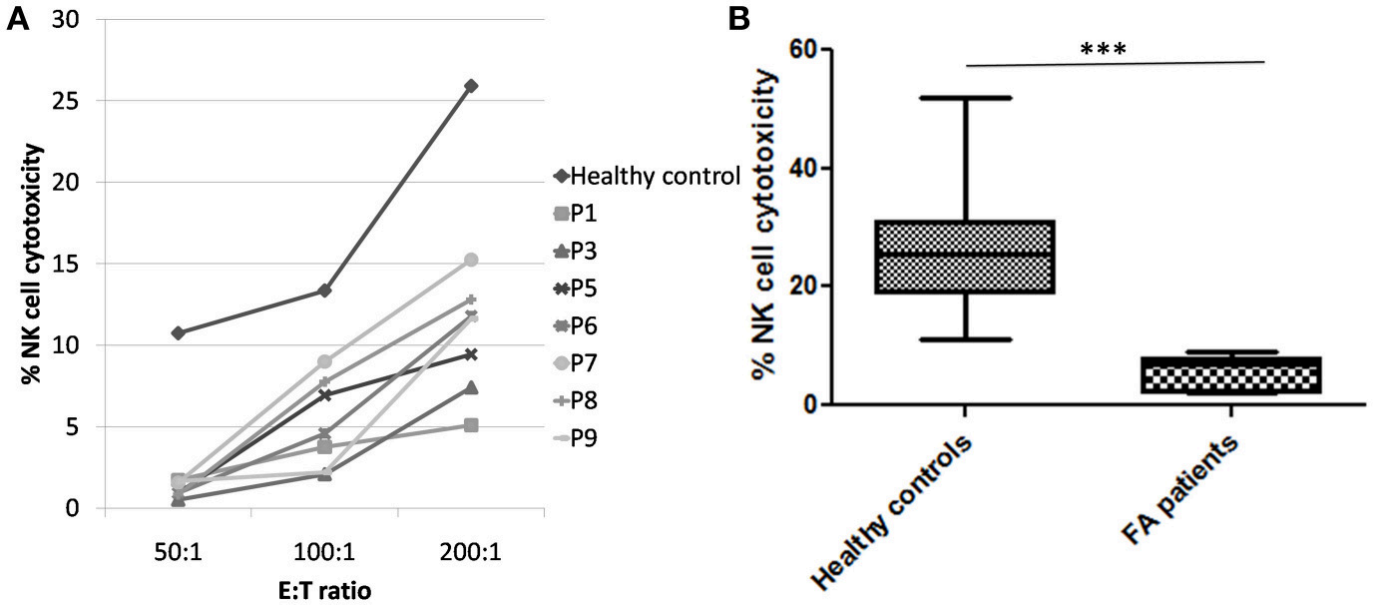
NK cell cytotoxicity in FA patients **(A)** Comparison of NK cell cytotoxicity in healthy control (median of all healthy controls processed) and FA patients at different E:T ratio (50:1, 100:1, and 200:1) FA patients had significantly low NK cell cytotoxicity compared to the healthy controls. **(B)** Comparison of NK cell cytotoxicity in healthy controls and FA patients at E:T ratio 200:1. Box-and-whiskers graph. The box extends from the minimum to the maximum and the line at the middle is the median. Mann-Whitney U test was used to evaluate differences (***P < 0.01).

Perforin expression on NK cells in all these patients was within the reference range (>65%; reference range 72 ± 2%) (Supplementary Figure 2). However, all patients had abnormal GRA on the NK cells (<15%; reference range 28 ± 8%) (*p* < 0.0001) (Figure 1) and had NK cell cytotoxicity lower than compared to the healthy controls (Table 2) (*p* < 0.0001; Figure 2; Supplementary Figure 3).

### Comparison of Cytokine Levels in FA and HLH Patients

**Figure 3:**
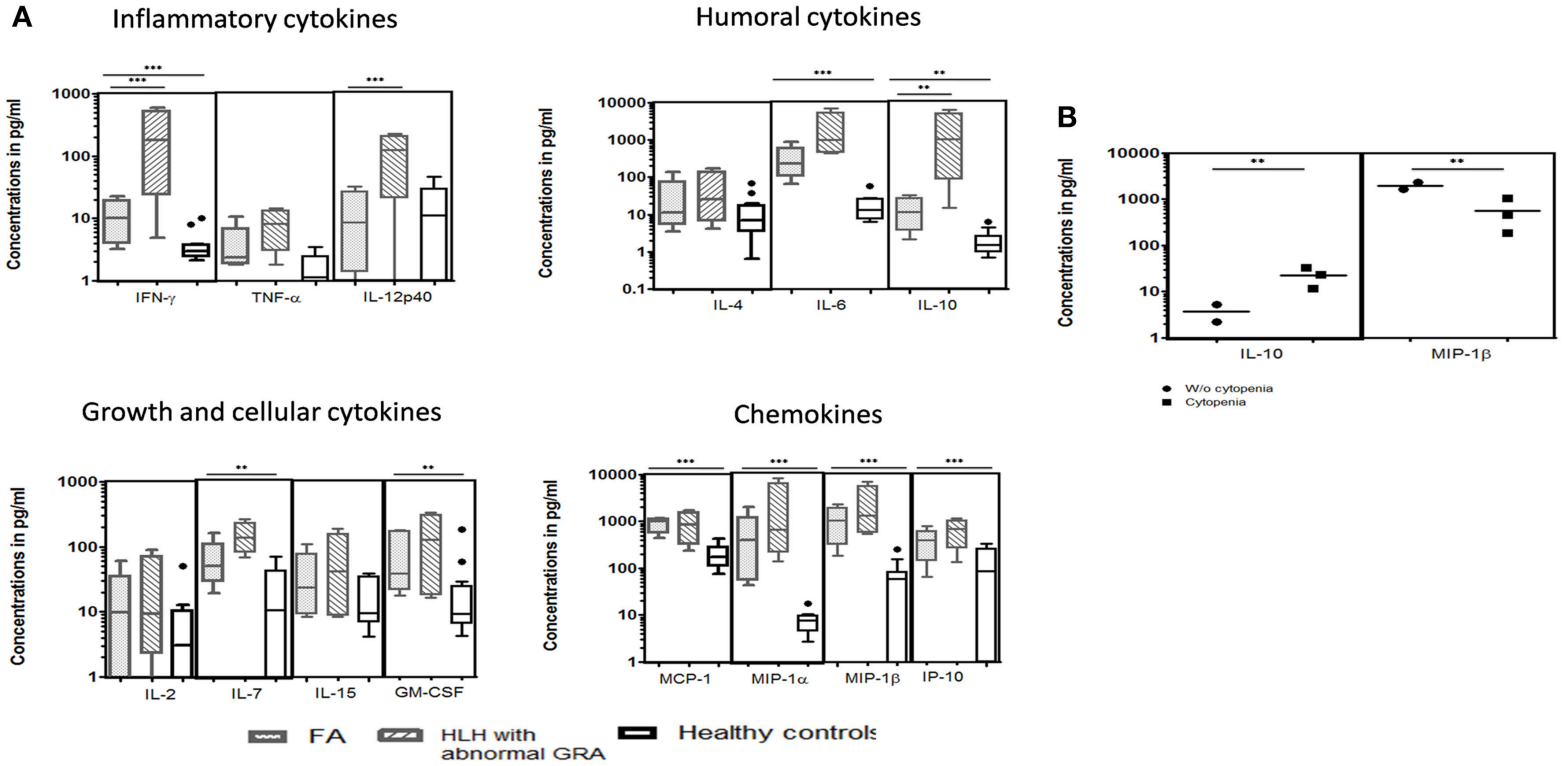
Comparison of serum cytokine levels Box-and-whiskers graph and the line at the middle is the median. **P < 0.05, ***P < 0.01. **(A)** FA patients (*n* = 5), HLH patients with abnormal CD107a expression on stimulated NK cells (GRA) (*n* = 13) and healthy controls (*n* = 12). Mann-Whitney U test was used to evaluate differences between cytokine concentrations in different groups. **(B)** FA patients with mild and severe cytopenia. Unpaired *t*-test was used to evaluate differences between cytokine concentrations in different groups.

We could perform cytokine levels in five patients (P1-5). Compared to the healthy controls, FA patients had significantly elevated IFN-γ, IL-6, IL-7, IL-10, GM-CSF, MCP-1, MIP-1α, MIP-1β, and IP-10 levels (Figure 3A). In FHL patients, the defect in granule release mechanism leads to an inefficient clearing of the infecting cells, causing hypercytokinemia. Thus, to understand whether the defective granule release mechanism in FA patients also had a similar effect, we compared the cytokine pattern of FA patients with FHL patients who had defective granule release mechanisms (GRA on NK cells: <15%; reference range: 28 ± 8%). It was observed that FHL patients had significantly elevated IFN-γ, IL-12p40, and IL-10 levels. No significant difference was seen in the cytokine pattern of FHL and FA patients with HLH. Interestingly, when FA patients with and without cytopenia were compared, it was observed that patients with cytopenia had significantly elevated IL-10 levels but reduced MIP-1β levels (Figure 3B).

### Viral Workup in FA Patients

Viral workup by real time PCR was negative in all FA patients included in the study.

### Genetic Findings

Molecular characterization was performed in five patients (P1-5) and revealed two novel mutations in the *FANCA* gene (P1, P2), one reported a mutation in the *FANCG* gene (P3), one a reported mutation in the *FANCL* gene (P5) and one patient had a compound heterozygous mutation in the *FANCN* gene (P4). None of these patients had a mutation in HLH related genes (viz. *PRF1, UNC13D, STX11, STXBP2, XIAP, SAP, LYST, RAB27A, AP3B1*).

The *in-silico* analysis of these variants was predicted to be damaging by a Mutation Taster2 (18). The detailed genetic diagnosis is presented in Table 1.

### Treatment and Outcome

Although all FA patients had abnormal GRA, the disease severity and response to therapy differed. Amongst the nine FA patients, patient P1 and P2 fit into the HLH criteria of the Histiocyte society. In view of the defective granule release assay (GRA), patient P1 was thought to be familial HLH and received Dexamethasone (10 mg) immediately along with danazol. The patient responded well with improvement in cell counts and reduced ferritin levels (64 ng/ml) and the Dexamethasone dose was tapered and stopped after 2 months. Patient P2 also fit into the HLH criteria, had abnormal GRA and was also diagnosed with FA. However, this patient was symptomatically treated and did not receive HLH targeted therapy. His cytopenia worsened and the patient succumbed to disease. Patients P3, P4, P6-9 had abnormal GRA but did not fit into the HLH criteria; cytopenia. These patients were treated with androgen therapy as per the FA treatment regime; and responded well. Interestingly, patient P5 also had abnormal GRA but did not fit into the HLH criteria. However, the patient presented with mild cytopenia and molecular studies revealed a mutation in the *FANCL* gene, confirming FA diagnosis. This patient was treated with danazol, and responded well-initially; however, the patients' cell counts started dropping further and they also presented purpura patches. In view of this, a HLH work-up was performed which revealed borderline elevated ferritin levels (642 ng/ml), few hemophagocytes were seen on the bone marrow aspirate and Dexamethasone (4 mg) was started, to which the patient responded well. This patient received Dexamethasone until late August 2018 and remains stable.

## Discussion

Fanconi anemia (FA) is a rare inherited disease characterized by congenital dysmorphic features, progressive bone marrow failure and increased risk of cancer development. In literature, a decreased NK cell count and/or impaired NK cell activity along with other immunological abnormalities have been described in patients with FA (1). However, to the best of our knowledge, this is the first report showing a defective degranulation mechanism leading to abnormal NK-cell cytotoxicity in FA-patients, which may explain the development of hyperinflammatory response in these patients.

Defective degranulation is classically described in patients with familial HLH (FHL) due to a genetic defect in one of the three genes; *UNC13D, STX11, STXBP2* and in HLH patients with hypopigmentation resulting from a genetic defect in *LYST, RAB27a, AP3B1, and AP3D1* genes (19). Patients with FHL typically present with a persistent high-grade fever, progressive cytopenias and hepatosplenomegaly (20). In some rare cases, organ specific manifestations like hepatitis, aplastic anemia/ isolated cytopenia and neurological manifestations may be seen in the absence of classic symptoms (21, 22). In this study, we evaluated nine FA patients, for a NK cell degranulation pattern, NK cell cytotoxicity and their cytokine profile; and compared it with FHL patients with a known degranulation defect. Two of these patients fit into the HLH criteria, and the remaining patients presented mild cytopenia without the classic clinical presentation of HLH.

All the patients included in this study had defective NK-cell degranulation, i.e., CD107a expression on stimulated NK cells was significantly low (<15%; reference range 28 ± 8%) compared to the healthy controls (*p* < 0.0001; Figure 1B); however, molecular studies revealed FA complementation (FANC) gene mutations and none had a mutation in HLH related genes (viz. *PRF1, UNC13D, STX11, STXBP2, XIAP, SAP, LYST, RAB27A, AP3B1*) (Table 1). Thus, indicating that the defect in the granule release mechanism observed in these patients is not a result of defect in HLH related proteins. FANC genes that encode FA proteins are not only important for DNA repair but are also involved in many biochemical pathways associated with immune function (23). Recent studies have increasingly highlighted the importance of non-canonical functions of FA proteins to understand the highly heterogeneous clinical manifestations seen in FA patients (24). Also, inflammatory pathology in FA has previously been reported (9, 24).

Although studies directly linking granule trafficking abnormality to any of the FANC genes are not available, we found some evidence in the literature for their possible role. It has previously been shown that the *FANC protein* is a multi-subunit complex composed of *FANCA, FANCC, FANCF*, and *FANCG* proteins. It interacts with some of the non-FA proteins such as the Sorting Nexin protein (SNX5), Brahma-Related Gene 1 protein (BRG1), IκB Kinase-2 (IKK2), Breast Cancer 1 (BRCA1) and is involved in various functions (25–28). Of these, SNX5 is a *FANCA*-binding protein and it has been proposed that the interaction of the FANCA protein complex with SNX5 may be involved in its subcellular trafficking (28). The results of experiments performed by Merino Trigo et al. (29) indicated that SNX5 may have functions not only associated with endosomal sorting but also with the phosphoinositide (PI)-signaling pathway (29). In 2008, Zebedin E reported that efficient degranulation of NK cells is absolutely dependent on PI3Kδ (30) which is a component of the phosphoinositide-signaling pathway. This conclusion was based on two independent sets of experiments by Eva Zebedin et al. (30), namely the externalization of the granule membrane constituent CD107a and the change in membrane capacitance associated with exocytosis. We suspect that the PI signaling pathway is an important link to explain the abnormal NK cell degranulation seen in all our FA patients. However, this still needs to be studied further.

Previous studies on immunological findings of FA patients had varied results with regards to the absolute counts of lymphocyte subsets. In a study by Myers et al. (11) and Korthof et al. (31), they found a reduced number of B and NK cell counts and normal T cells in FA compared to the control group, whereas, Giri et al reported low T, B, NK cells in FA (32). In all these studies, the majority of patients had lower NK cell counts. In our study as well, lymphocyte subset analysis revealed low NK cell counts in seven out of the nine patients evaluated. This variation in the findings in different studies might be attributed to the difference in the phase of the disease and the age of patients included in the various studies.

NK cells are critical in modulating the initial response of antigen-presenting cells to invading pathogens and attenuate the subsequent activation of antigen specific T cells especially cytotoxic T cells (CTL). This failure to attenuate the activation of the immune response leads to tissue infiltration by CTL and macrophages, resulting in the release of pro-inflammatory cytokines including IFN–γ, causing persistent macrophage activation (19, 33). In HLH, impaired NK cell function causes immune dysregulation, leading to hypercytokinemia which in turn leads to pancytopenia, hyperferritinemia, hypertriglyceridemia etc. In FA patients elevated levels of cytokines viz. IFN–γ and TNF-α has previously been reported (34). However, the mechanism for overproduction of these cytokines remains unclear and is considered to be a response to bone marrow dysfunction, chronic inflammation and tissue injury (9, 10, 35). We evaluated various cytokines essential for the inflammatory response, initiating humoral response, cellular growth and chemokines in FA patients and compared the cytokine pattern with FHL patients that have a degranulation defect, to understand the underlying mechanism. Though the pathogenesis of FHL and FA differs, interestingly, the cytokine pattern was found to be similar. Compared to the healthy controls, both the FA and FHL patients had significantly elevated IFN-γ, IL-6, IL-10, IL-7 GM-CSF, MCP-1, MIP-1α, MIP-1β, and IP10/CXCL10 levels. However, the degree of elevation of IFN-γ and IL-10 was much more in patients with FHL compared to FA patients. IFN-γ is a Th1 cytokine that is produced in response to inflammatory stimuli, to regulate the human immune response. IL-10, on the other hand, has strong immunosuppressive properties and acts as a potent inhibitor of Th1 effector mechanisms. The level of these cytokines determines immune regulation and their overproduction plays an important role in the pathophysiology of FHL (36). Therefore, despite a similar cytokine pattern in FA and FHL patients, a relatively higher degree of hyperinflammatory response observed in FHL patients may be attributed to the higher levels of IFN-γ and IL-10.

So, in both FHL and FA there is hyperinflammation, hypercytokinemia and NK cell dysfunction. In FHL, it is the NK cell dysfunction that causes the inability to clear the antigenic stimulus and failure to terminate the inflammatory response, ultimately leading to hypercytokinemia and a hyperinflammatory immune response (16). However, in FA the mechanism is still not clear. One potential mechanism could be the same as that seen in FHL, i.e. NK cell dysfunction causing hypercytokinemia and hyperinflammation. However, it is known that cytokines can drive NK cell differentiation and induce NK cell dysfunction (37–39). Also, macrophage dysfunction is known in FA cells (40–42) and cytokines secreted by macrophages have been reported to inhibit NK cell function (43). Thus, another potential mechanism may be considered vice versa, i.e., NK cell dysfunction is secondary to ongoing macrophage-promoted inflammatory processes seen in FA patients. Distinguishing between these two possibilities is beyond the scope of this work and requires further *in-vitro* experiments and animal studies.

FA patients presenting severe cytopenia had elevated IL-10 levels while having reduced MIP-1β levels compared to FA patients with mild cytopenia. IL-10 is known to induce thrombocytopenia and anemia (44, 45) and it also inhibits human mononuclear cell expression of MIP-1 proteins; both MIP-1α and MIP-1β (46). Inflammatory responses elicited by MIP-1α and MIP-1β vary between complementary and antagonistic actions. MIP-1α has the potential of inhibiting early hematopoietic stem cell proliferation; whereas MIP-1β in excess can block the MIP1α effects (47). In FA patients, both MIP-1α and MIP-1β were elevated; however, FA patients with mild cytopenia had higher MIP-1β levels compared to FA patients with severe cytopenia. High levels of MIP-1β might suppres the MIP1α inhibitory effect on early hematopoietic stem cell proliferation which may contribute to maintaining cell counts. However, this needs to be studied in a larger cohort of FA patients.

Viral infections are one of the most common triggering factors for developing HLH. EBV (Epstein-Barr virus) is the most commonly reported triggering pathogen, especially in Asia. Other viruses identified as triggers of HLH are human herpes virus 6, cytomegalovirus, adenovirus, parvovirus, and varicella-zoster virus (48). The possibility of viral infection induced development of HLH in FA patients was ruled out by viral PCR. This indicated that HLH and hyperinflammatory responses in these patients was not triggered by a viral infection.

Despite significant advances in the understanding of the pathophysiology of FA, the management options available are few and remains challenging. The only definitive option available to date is HSC transplantation (HSCT). However, this is also associated with an increased risk of developing malignancies, especially with unrelated donors (23). Thus, transplantation is reserved for patients with moderate to severe cytopenia, rapidly deteriorating hematopoietic function and development of MDS or AML and dependent on the availability of a suitable matched donor. Androgens (Danazol and Oxymetholone) remain the drug of choice for mild to moderate cytopenias, however it cannot be continued for a long time due to the development of cytogenetic abnormalities, lack of response, liver adenomas and the development of AML/MDS (49). In the present study, two patients presented a clinical diagnosis of HLH, and P1 was started on the HLH 2004 protocol (including Dexamethasone and cyclosporine) and responded well but, patient P2 who was treated with only supportive management succumbed to the disease. Patients P3, P4, P6, P7, P8, and P9 presented cytopenia and responded well to androgen therapy. Patient P5 initially presented mild cytopenia and was treated with androgen therapy, however, later in view of dropping cell counts, Dexamethasone (4 mg) was started, to which the patient responded well. This highlights the need for early suspicion and evaluation for HLH, especially in a setting of rapidly deteriorating cytopenias in patients with FA and the early initiation of the immunosuppressive therapy in these selected patients. A combined US-Italian study showed marginal improvement in counts of FA patients with anti-TNF-α and the authors proposed better anti-inflammatory therapy for FA (50). Novel targeted therapeutic approaches like the anti-IFN-γ monoclonal antibody, JAK1/2 inhibitor which is shown to be effective in primary HLH (51, 52) can be considered in patients with FA especially with elevated cytokine levels.

Overall, this study highlights NK cell degranulation defects, hypercytokinemia and susceptibility to developing HLH as one of the important mechanisms for cytopenias in FA. FA patients with clinical features such as a persistent high-grade fever, rapidly progressing cytopenias and organomegaly must be evaluated for HLH. Early diagnosis and initiation of HLH directed therapy, may lead to a better outcome. This study also proposes that FA genes are included in the list of genes for familial HLH. Further studies, however, are required to understand the role of FA genes in the impairment of granule trafficking in different cell types and also to understand the mechanism of NK cell dysfunction in FA patients.

## Ethics Statement

This study was approved by the Institutional Ethics committee for Human subjects (ICMR-NIIH) and was carried out after obtaining informed consent from the FA patients/ parents.

## Author Contributions

SnS analyzed the data and wrote the manuscript. MK, NC, and MG were involved in performing the laboratory investigations. PN, MD, UB, PM, AC, and CS supervised the clinical care of the patients. VS provided the viral work-up data. MM and BV supervised the study and reviewed the manuscript.

### Conflict of Interest Statement

The authors declare that the research was conducted in the absence of any commercial or financial relationships that could be construed as a potential conflict of interest.

## Supplementary Material

Supplementary Figure 1 Microscopic images of chromosomal Breakage. **(A)** Representative image of a healthy control. **(B–D)** Representative images of three FA patients. Arrow indicates the chromosome breaks seen in the patient.

Supplementary Figure 2 Perforin expression on NK cells in FA patients: **(A)** Samples were analyzed by flow cytometry, gating on lymphocytes by forward/side scatter. Perforin expression was analyzed on natural killer (NK) cells (CD56+CD3-)(Dark blue dots) using T helper cells as internal negative controls (Light blue dots) **(B)** Perforin expression results are shown from a healthy control (representative plot) and patients with FA included in this study.

Supplementary Figure 3 NK cell cytotoxicity by flowcytometry at different E:T ratio (50:1, 100:1 and 200:1) **(A)** Gating of K562 cells based on FSC-SSC scatter **(B)** unlabeled K562 cells **(C)** DIOC18 dye (FITC) labeled K562 cells **(D)** Spontaneous K562 cells death **(E)** NK cell cytotoxicity in healthy control (representative plot) **(F–L)** NK cell cytotoxicity in FA patients. The percentage of NK cell cytotoxicity is indicated on each plot.
