# Supplementary figures and images for: Natural Killer Cell Degranulation Defect: A Cause for Impaired NK-Cell Cytotoxicity and Hyperinflammation in Fanconi Anemia Patients
Source: Front Immunol. 2019 Mar 21;10:490. doi: 10.3389/fimmu.2019.00490 (PMC6438155; doi:10.3389/fimmu.2019.00490)

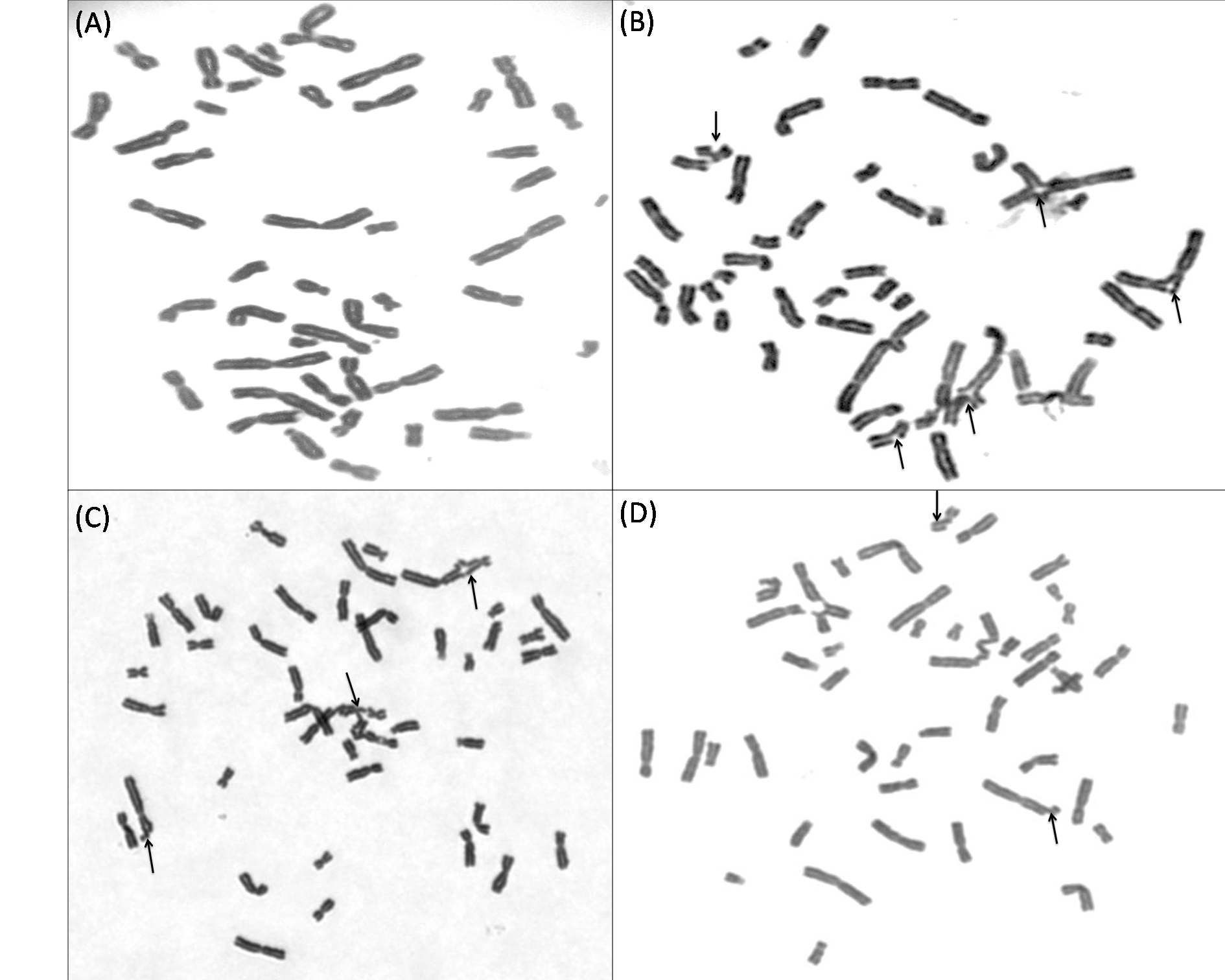

Supplement: Supplementary Figure 1 — Microscopic images of chromosomal Breakage. (A) Representative image of a healthy control. (B–D) Representative images of three FA patients. Arrow indicates the chromosome breaks seen in the patient. [file Image_1.TIF]

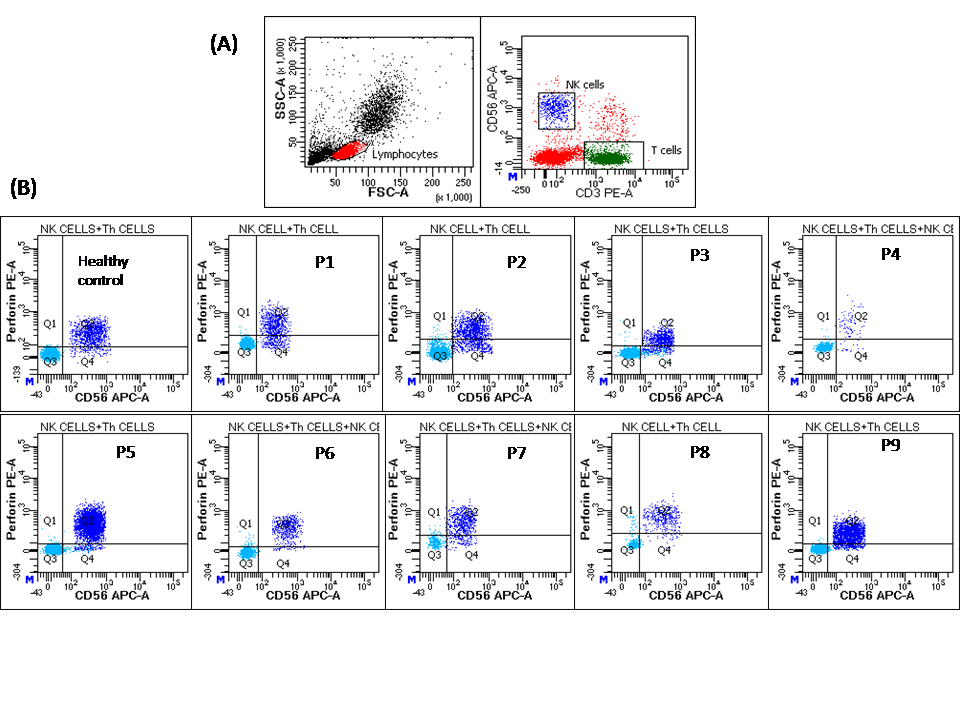

Supplement: Supplementary Figure 2 — Perforin expression on NK cells in FA patients: (A) Samples were analyzed by flow cytometry, gating on lymphocytes by forward/side scatter. Perforin expression was analyzed on natural killer (NK) cells (CD56+CD3-)(Dark blue dots) using T helper cells as internal negative controls (Light blue dots) (B) Perforin expression results are shown from a healthy control (representative plot) and patients with FA included in this study. [file Image_2.TIF]

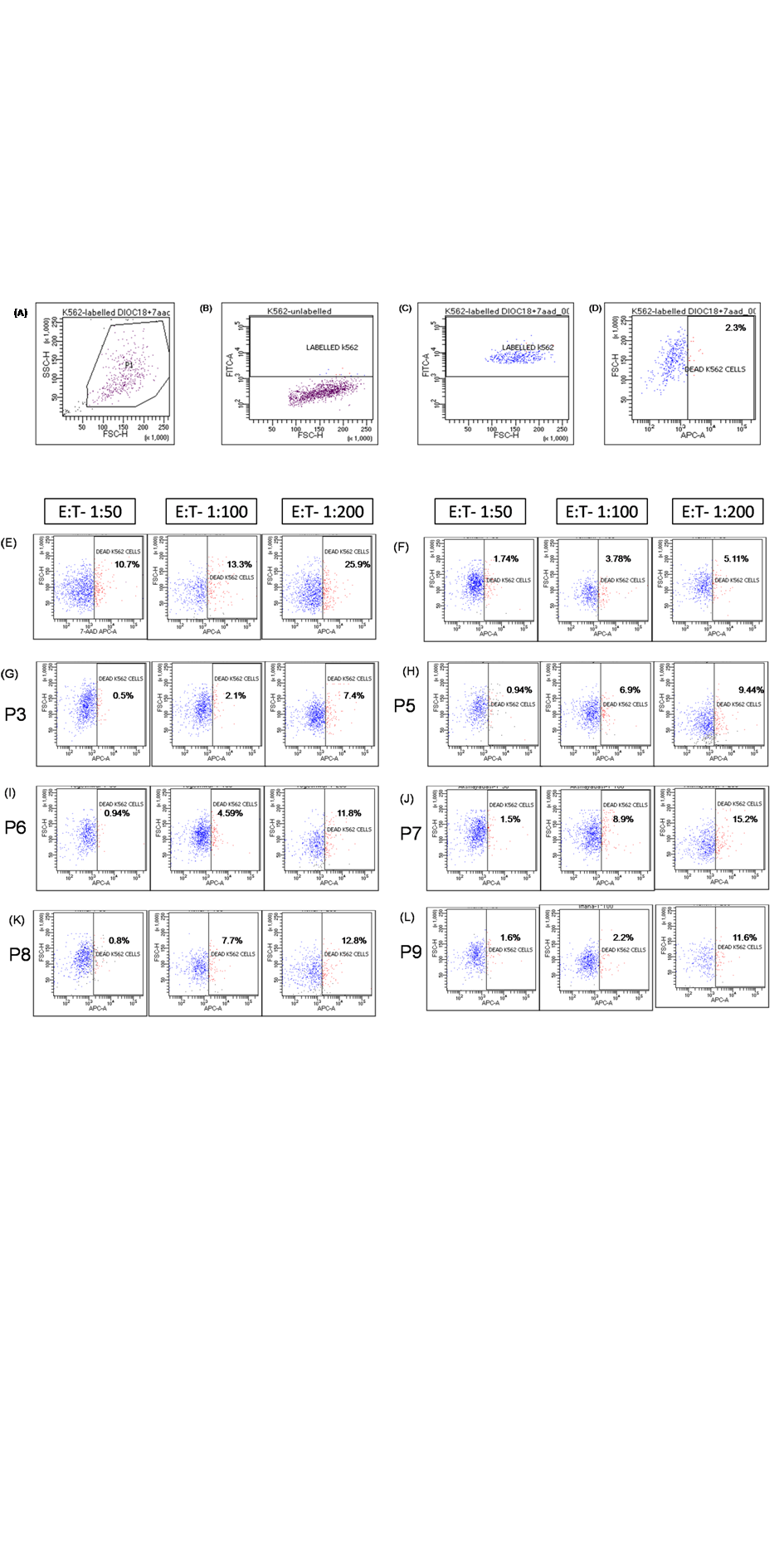

Supplement: Supplementary Figure 3 — NK cell cytotoxicity by flowcytometry at different E:T ratio (50:1, 100:1 and 200:1) (A) Gating of K562 cells based on FSC-SSC scatter (B) unlabeled K562 cells (C) DIOC18 dye (FITC) labeled K562 cells (D) Spontaneous K562 cells death (E) NK cell cytotoxicity in healthy control (representative plot) (F–L) NK cell cytotoxicity in FA patients. The percentage of NK cell cytotoxicity is indicated on each plot. [file Image_3.tif]
